# Depression Assessment in the German National Cohort (NAKO)

**DOI:** 10.1093/eurpub/ckac129.027

**Published:** 2022-10-25

**Authors:** K Berger, M Rietschel, F Streit

**Affiliations:** 1 Institute of Epidemiology and Social Medicine, University of Münster, Münster, Germany; 2 Medical Faculty Mannheim, University of Heidelberg, Heidelberg, Germany

## Abstract

**Background:**

The mental health status of populations (public mental health) and its effect on societies has gained considerable attention in recent years, especially during the current pandemic. The measurement of depressive symptoms is at core of the assessment of mental health. The detailedness of this assessment defines the range of public mental health problems that can be answered.

**Methods:**

Between 2014 and 2019 the German National Cohort (NAKO) recruited 205,000 participants aged 20-70 years into the baseline examination in 18 study centers. Depression and depressive symptoms were assessed by different instruments including a lifetime diagnosis of depression and current treatment, the Major Depressive Disorder (MDD) section of the Mini-International-Neuropsychiatric-Interview (M.I.N.I. 5.0) and the depression scale of the Patient Health Questionnaire (PHQ-9). These instruments include different time periods and interpretations. Associations between these depression outcomes and age, gender and education are analysed in linear and logistic regression models.

**Results:**

A lifetime physician's diagnosis of depression was reported by 14.7% of participants with considerable regional variation and almost 50% of this group received treatment within the last 12 months. Based on PHQ-9 7.9% of the participants were classified as depressive according to the dimensional assessment (score≥10) and 3% of them as having a major depression subtype. In contrast 32.8% screened positive based on the MINI and 15.4% of those receiving the full MINI had a diagnosis MDD. Associations with important socioeconomic determinants for these different depression outcomes will be reported.

**Conclusions:**

The large NAKO sample size and the detailed assessment of depression symptoms and status enables the analysis of a broad range of public mental health questions. The analysis of depression frequencies and the distribution of depressive symptoms allow the establishment of population references.

